# Contrast-enhanced Ultrasound in a Case of Acute Renal Artery Occlusion

**DOI:** 10.5334/jbsr.1383

**Published:** 2018-02-20

**Authors:** Marie-Sofie Walgraeve, Luc Steyaert, Katrien Gieraerts

**Affiliations:** 1 AZ Sint-Jan Brugge-Oostende AV, BE

**Keywords:** Contrast enhanced ultrasound, Diagnostic imaging, Renal artery occlusion, Patient safety

## Case Presentation

A 64-year-old man presented at the emergency department with colic pain in the left lumbar region. The pain started acutely and was ongoing for more than one day. There were no urinary complaints, no vomiting, no respiratory complaints and normal stools. His medical history showed a lung carcinoma for which he was currently being treated with chemotherapy, hypercholesterolemia and a compression fracture of D4. On clinical examination, there was a left costovertebral angle tenderness. Urinary analysis was negative for proteins, glucose, bilirubin, hemoglobin, red and white blood cells. Laboratory results showed only slightly elevated C-reactive protein of 5.4 mg/L (<5 mg/L), slightly decreased hemoglobin of 12.1 g/dl (13.1–17.2 g/dL), elevated urate of 71 mg/dl (16.6–48.5 ml/dL), elevated creatinine of 1.6 mg/dL (0.67–1.17) and estimated glomerular filtration rate > 60 mL/min/1.73m^2^.

B-mode abdominal ultrasound revealed normal anatomy of both kidneys and the bladder wall.

**Figure 1:**
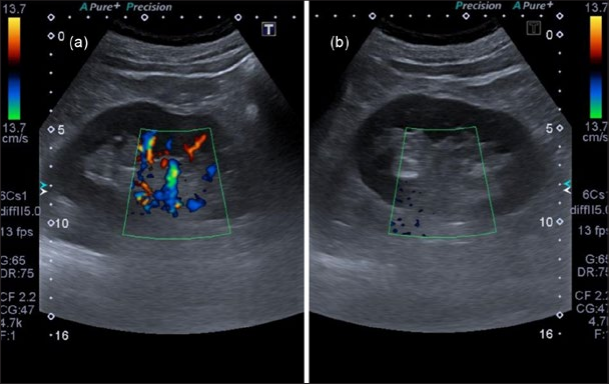
Doppler-mode ultrasound of right and left kidney. **1a:** Normal vascular signal at the hilum of the right kidney. **1b:** Absent vascular signal at the hilum of the left kidney with the same ultrasound parameters.

**Figure 2:**
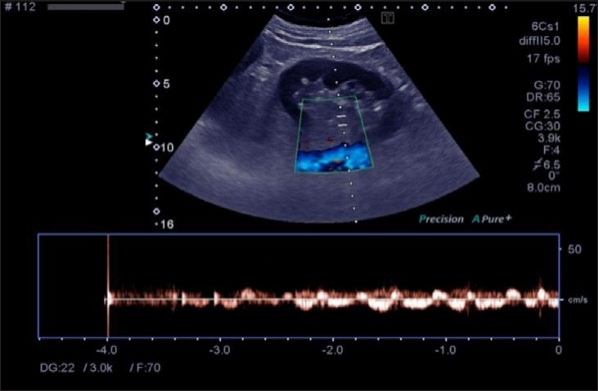
Weak, alternating venous flow at the left kidney hilum, indicating venous stasis.

Unenhanced abdominal computed tomography (CT) showed no abnormalities. The patient was admitted for intravenous pain management and observation. Pain persisted and a follow-up abdominal ultrasound was performed. The kidneys remained morphologically normal on B-mode. Color Doppler ultrasound showed normal vascularization of the right kidney (Figure 1a). There was no arterial signal in the kidney hilum or in the renal cortex of the left kidney (Figure 1b) that exhibited only weak, alternating venous flow (Figure 2). These findings were highly suspicious of left renal artery occlusion.

**Figure 3:**
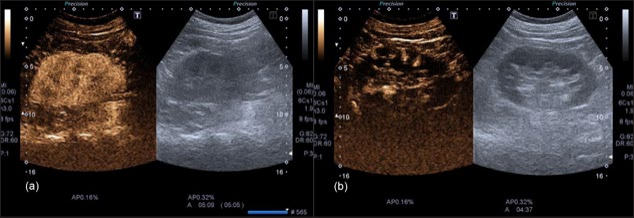
Contrast enhanced ultrasound of the kidneys. **3a:** Normal vascular supply of the right kidney with homogeneous enhancement of the cortex. **3b:** Minimal cortical enhancement of the left renal cortex, extending from the periphery to the hilum, representing perforating branches of the capsular artery, also known as rim sign, indicative of renal infarction.

Because of progressively decreasing kidney function, a contrast-enhanced ultrasound (CEUS) was performed. This showed normal vascular supply of the right kidney with homogeneous enhancement of the cortex (Figure 3a). On the left, there was only minimal cortical enhancement which extended from the periphery to the hilum, representing perforating branches of the renal capsular artery (Figure 3b). This finding is also known as the cortical rim sign which is seen on contrast-enhanced CT- or Magnetic Resonance-images in case of renal infarction. The findings confirmed an occlusion of the left renal artery.

Because complaints started more than 72 hours prior to diagnosis, there was no indication for thrombolysis. Therapeutic doses of low molecular weight heparines were started to prevent new thrombotic events and an adequate level of pain killers was continued. Kidney function increased over the next few days with eGFR of 53 mL/min/1.73m^2^ and creatinine of 1.4 mg/dL at hospital discharge.

## Comment

Ultrasound is the preferred imaging modality in patients with known or suspected renal disease. B-mode ultrasound is used to measure renal size and to detect focal lesions and obstruction of the collecting system. Color Doppler ultrasound can be added to look for vascular disorders. However, it is limited by attenuation, poor sensitivity for slow blood flow and angle dependency. Contrast-enhanced ultrasound adds valuable information in an accurate and relatively simple way. Because of the high vascularization of the kidneys and the ability of ultrasound contrast agents to visualize very small vessels, both evaluation of focal kidney lesions and global renal vascularization are possible. Because it is not excreted by the kidneys, CEUS can be used in acute or chronic renal failure [1,2]. Also, it can be safely used in patients with contrast allergy [2].

CEUS may be useful to detect or confirm the diagnosis of renal infarction. Several investigations, both in animals and in humans, have shown excellent diagnostic performance in the detection of renal parenchymal ischaemia, similar to that of contrast-enhanced CT and superior to color Doppler ultrasound [2]. Because of the excellent spatial resolution of CEUS, confident differentiation between focal cortical necrosis – wedge-shaped non-enhancing cortical areas with preserved hilar vascularity – and renal infarction – nonperfusion of the whole kidney (e.g. in this case) – can be made [1,2]. CEUS can show the cortical rim sign that arises after renal infarction due to collateral capsular perfusion. Another advantage of CEUS is the possibility to differentiate between non-perfused, infarcted tissue and hypoperfused parenchymal regions [2]. On color Doppler ultrasound both appear as areas lacking colour signal while on CEUS only infarcted areas completely lack contrast enhancement after microbubble injection. The European Federation of Societies for Ultrasound in Medicine and Biology (EFSUMB) recommends CEUS in case of suspected vascular renal disorders, including renal infarction and cortical necrosis (recommendation level A, 1a) [2].
